# Responsive Nano-structured Cubosomes: Advancements and Therapeutic Applications

**DOI:** 10.34172/apb.025.43330

**Published:** 2025-05-31

**Authors:** Ishal Miranda, Biyas Misra, Manasa Chikballapur Manjunath, Geetha Nayak, Ullal Likhitha, Usha Yogendra Nayak

**Affiliations:** Department of Pharmaceutics, Manipal College of Pharmaceutical Sciences, Manipal Academy of Higher Education, Manipal 576104, Karnataka, India

**Keywords:** Nanoparticles, Targeted drug delivery, Drug encapsulation, Self-assembly, Multi-stimuli Responsive cubosome, pH-sensitive nanoparticles

## Abstract

Nanotechnology has revolutionized drug delivery, which offers innovative ways to maximize treatment efficacy while decreasing side effects. The lyotropic liquid crystalline nanoparticles (LLCNP), such as cubosomes and hexosomes, have gained substantial interest because of their distinctive molecular arrangements. Lipophilic, hydrophilic, and amphiphilic drugs can be encapsulated by cubosomes, making them versatile carriers in drug delivery systems. Different types of cubosomes, such as pH-responsive, temperature-responsive, light-responsive, enzyme-responsive, and multi-stimuli-responsive, have been discussed in this review detailing their preparation methods and therapeutic applications. Cubosomes possess high surface area, are biocompatible, and provide enhanced drug protection. However, formulation stability and scalability are the main challenges. This paper highlights the potential of cubosomes for targeted drug delivery, focusing on their ability to optimize bioavailability and controlled drug release.

## Introduction

 Nanotechnology has developed drug delivery by providing novel solutions that improve treatment outcomes while reducing adverse effects. Active pharmaceutical ingredients characterized by extremely low solubility present restricted bioavailability upon oral ingestion, diminished diffusion capacity via the outer membrane, the necessity for higher quantities during intravenous administration, and undesired side effects preceding the conventional formulation methods.^1^ Integrating nanotechnology in drug delivery mechanisms offers a pathway to overcome these constraints. Lyotropic liquid crystalline nanoparticles (LLCNP) emerge as exemplars of self-assembling nanomaterials, showcasing remarkable potential.^2-5^ Among the array of nanostructured carriers, cubosomes epitomize a burgeoning class of nanosystems engineered to accommodate various active pharmaceutical ingredients, consisting of both hydrophobic and hydrophilic drugs, alongside biotherapeutics such as peptides, proteins, and nucleic acids.^6-9^

**Figure 1:**
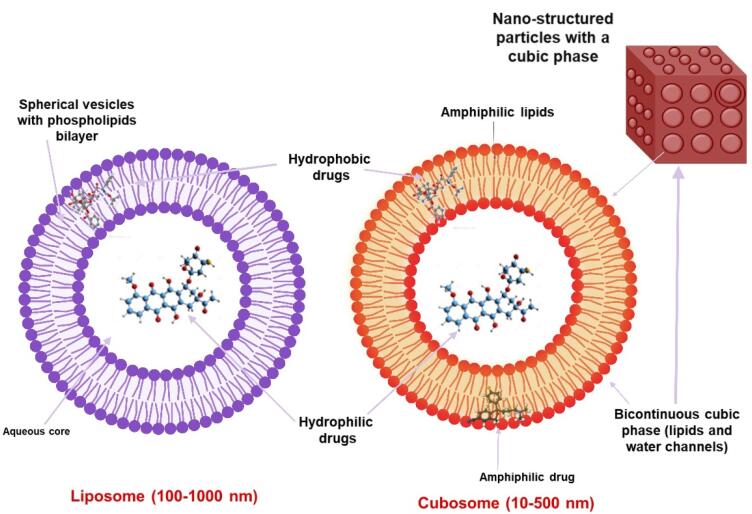
The distinction between liposomes and cubosomes is illustrated here. Created in BioRender. https://BioRender.com/kh1in0u

 The process of liquid crystalline injectable formulations involves integrating amphiphilic molecules within a solvent, resulting in the formation of LLC phases. The amphiphilic structure of the molecule, additives, and the solution’s conditions affect these phases.^10^ Friedrich Reinitzer made the first observation of LLCNP in 1999.^7^ Like liposomes, they have intricate 2D and 3D nonlamellar nanostructures, including inverse hexagonal and cubic mesophases. The distinctions between liposomes and cubosomes are illustrated in Figure 1.

 Cubosomes are square and rounded shapes and possess an internal cubic lattice. They are thermodynamically stable structures characterized by honeycombed SL formulations that create two internal aqueous channels and a substantial interfacial area.^11,12^ Inside the bicontinuous cubic stage framework, there are three stages [Im3m (Schwarz) surface), Pn3m (Diamond surface), Ia3d (gyro surface)] to explain the distinct shapes, all of which additionally display boosted drug transportation in the target site.^13-16^ According to Ayesha Waheed, the organized mesophase structure and nanoscale size range of liquid crystalline NPs make them useful drug carriers for molecules with different polarity, such as nucleic acids and proteins. A thorough analysis highlights how their 3D structure and tunable coronas support a variety of applications, from theranostics to medication delivery.^7^ Zhai et al reflected that liquid crystalline drug delivery systems are promising for the future generation of nanomedicine, with their self-assembling amphiphilic lipids acting as efficient nanocarriers for a variety of medications, peptides, proteins, nucleic acids, and imaging agents.^17^

 The unique cubic structure not only presents a platform for superior drug encapsulation and protection but also offers tailored solutions for targeted therapeutic interventions.^18-20^ Cubosomes possess high surface area and responsiveness to stimuli. Using this, researchers can design and tailor sophisticated delivery systems capable of precise control over drug release and distribution within the body.^21,22^ Moreover, the diversity of types of cubosome each with its own set of advantages and challenges, underscores the necessity of a comprehensive exploitation. We intend to inspire further research and innovation in the nanotechnology field, driving advancements in therapeutic interventions, and improving patient care.

## pH-Responsive cubosomes

 There are pH differences between normal blood and pathological tissues (e.g., those affected by infection, inflammation, and cancer, which often become more acidic), among specific intracellular compartments such as the cytosol, endosomes, and lysosomes, and along the gastrointestinal tract. These differences are typically targeted by pH-responsive nanosystems. For this scope, “smart” molecules including polymers, lipids, and peptides are used since they are biocompatible and sensitive to specific pH levels because of their functional ionizable groups.^23,24^

 The pH-responsive mesophase, designed with a lipid-based LLC system composed of mono-linolein and pyridinyl methyl linoleate, transitions its symmetry from a reverse hexagonal phase (H_2_) at pH 7.4 to a bi-continuous cubic phase (pn3m) at pH 5.5. This transformation was studied and found to occur due to the protonation of the pyridinyl methyl linoleate’s weakly basic head group near its pKa of 5.5.^25^ This pH-triggered behavior leverages the acidic conditions in tumor tissues to enhance the release of drugs like Doxorubicin, potentially improving chemotherapy efficacy. By switching its structure under acidic conditions, this mesophase enables targeted drug delivery, improving the efficiency of cancer treatments.^26,27^

 According to Rajesh et al, pH-responsive cubosomes lessen side effects while allowing a chemotherapeutic chemical to be delivered to tumors selectively.^28^ According to Mertins et al, pH-sensitive polymer shells present novel prospects for topical and oral medication delivery that could lead to the development of innovative cancer treatments. Drug compounds that have electrochemical activity might also be advantageous for pH-responsive drug release.^4^ At physiological pH, these NPs had a slow-releasing hexagonal structure, whereas, at the acidic pH of the tumor, they displayed a quick-releasing bi-continuous cubic phase.^29^

**Figure 2:**
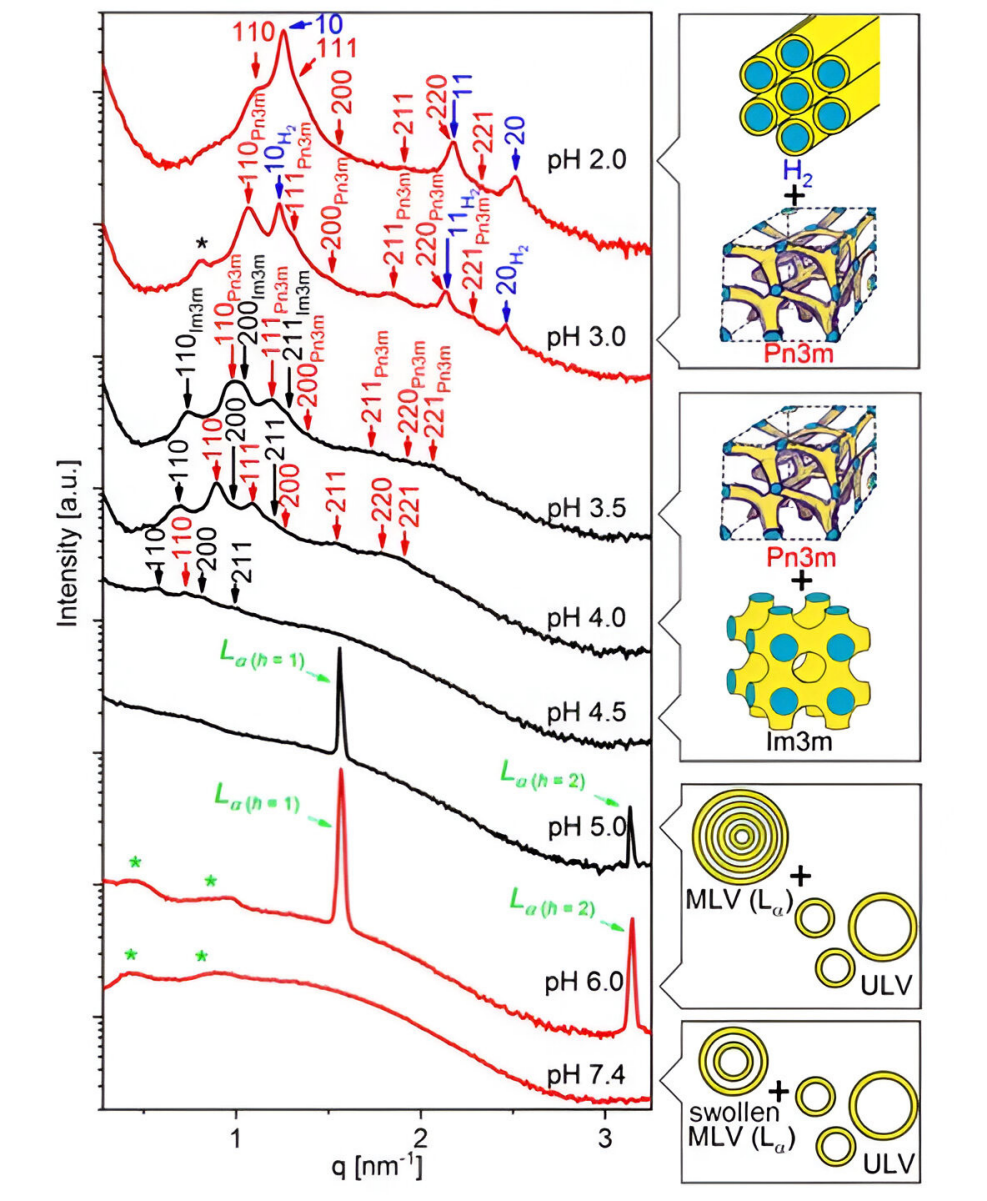
Small-angle X-ray scattering (SAXS) patterns were collected for polymer-stabilized, pH-sensitive NPs containing the anticancer drug 2-hydroxyoleic acid. The red curves represent SAXS patterns as the pH decreases from neutral to very acidic, while the black curves show patterns as the pH increases back to 5. Pn3m (red arrows) and Im3m (black arrows) indicate cubic arrangements and H2 (blue arrows) is a hexagonal arrangement, illustrating structural changes in the NPs with varying pH levels. The figure is reprinted with permission from Prajapati et al.^34^ Copyright 2019, American Chemical Society

 According to Manchun et al, pH-sensitive nanosystems have been synthesized to deliver medications to the endosomes or lysosomes within cancer cells, or the mildly acidic extracellular fluids of tumor tissue following endocytosis. After the medication accumulates in tumor tissue via enhanced permeability and retention effect, these systems can release it through specialized mechanisms. Alternatively, they can release the drug within endosomes and lysosomes via pH-controlled hydrolysis after cellular uptake through the endocytic pathway.^29^ According to Negrini and Mezzenga, linoleic acid, a weak acid with a pKa of approximately 5, provides pH responsiveness. At pH 7, it is essentially in the deprotonated charged state, while at pH 2, it is primarily protonated and neutral. This results in changes to the critical packing parameter of the LLC.^30^ In the study by Prajapati et al pH-responsive cubosomes were synthesized by blending 2-Hydoxyoleic acid with glycerol monooleate at varying mass ratios to examine pH-induced structural transformation for targeted drug delivery to cancer tissues. The research aimed to investigate the composition and pH dependence of drug-loaded NPs, providing insights into their pH-triggered transformation (Figure 2).^31^

## Temperature responsive cubosomes

 Numerous investigations have corroborated the hypothesis that drug release from thermos-responsive polymers can be triggered by slight temperature variations. However, in recent years, thermos-responsive drug delivery systems have primarily been utilized in the delivery of anti-cancer drugs and imaging agents.^32^ Poloxamers stand out as widely explores thermo-responsive systems, renowned for their versatility. Thermo-responsive systems offer numerous advantages, such as the formulation of an *in-situ* gelling-controlled release system.^33^

 Mohsen et al elucidated the significance of a thermos-sensitive system in enhancing the intranasal delivery of Lamotrigine. By integrating optimized cubosomes into a thermosensitive in *situ gel*, the system enhances the physical stability, nasal residence time, and patient compliance. The thermos-sensitive gel undergoes a sol-gel transition in response to temperature changes, facilitating the administration as a liquid that transforms into a gel upon contact with the nasal mucosa. The transition augments drug absorption across the nasal mucosa membrane, thereby improving the efficacy of lamotrigine in epilepsy treatment. The thermosensitive property is attributed to the *in-situ* gel into which the optimized cubosomes are incorporated. The synergistic combination of cubosomes and the thermosensitive gel enhances drug absorption and efficacy by prolonging nasal residence time and facilitating drug release at the target site.^34^

 According to Dabkowska et al, the tiny poly(*N*-isopropylacrylamide) nanogels act as precise thermos-responsive controllers in regulating the hydration of liquid crystalline surface layers.

 Their rapid transition from a swollen to a collapsed state, induced by temperature changes, allows controlled release of water from the surface while preserving the integrity of the lipid matrix. This capability enables the secure encapsulation of delicate bioactive molecules within the lipid matrix, presenting a promising avenue for controlled-release applications.^35^

## Light responsive cubosome

**Figure 3:**
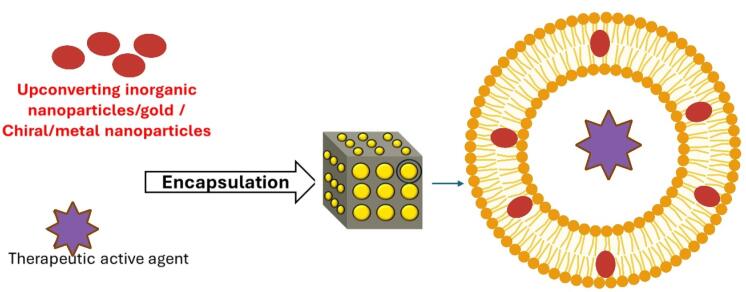
Preparation of light-responsive cubosomes including the encapsulation of upconverting inorganic NPs/gold/chiral/metal NPs. Plasmonic nanoparticles were co-encapsulated with an active agent within cubosomes to enhance the therapeutic capabilities of nanoplatforms. Created in BioRender. https://BioRender.com/yui5fdo

 Light-responsive nanocarriers offer a non-invasive, highly adaptable, and precisely controlled method for drug delivery.^36^ The advancement of stimuli-responsive materials is crucial, marking the initial utilization of photo-switchable amphiphiles for the creation of light-sensitive cubic LLC dispersions, known as cubosomes. This innovation enables external manipulation of the LLC structure, facilitating the on-demand release of entrapped guest molecules. To produce these cubosomes, azobenzene photo surfactants, which have an azobenzene-alkyl tail and a neutral tetra ethylene glycol head group, are used with monoolein-water systems.^37^ The lipid/water system can be made photo-responsive by including plasmonic NPs or photochromic compounds. When these components are activated, the lipid bilayer’s permeability changes either temporarily or completely, enabling or prohibiting the movement of molecules that are encapsulated (Figure 3). By using photothermal and photochromic techniques, this has been accomplished.^38^

 Chen et al highlighted that phototherapy, which includes photothermal therapy and photodynamic therapy, has attracted considerable interest among researchers because of its non-invasive nature, precise spatial and temporal selectivity, and low toxicity.^39^ As per Fong et al, plasmonic hydrophobized gold nanorods are integrated into mesophases produced by diverse lipid/water combinations to fabricate light-responsive bulk self-assembly lipid systems. The gold nanorods nestled within the liquid crystalline matrix were stimulated by Near Infrared laser light, inducing localized plasmonic heating of the mesophase. This facilitated the reversible manipulation of nanostructure, contingent upon the concentration of nanorods and the composition and heat capacity of the liquid crystalline matrix.^38^

 According to Angelova et al in host-guest LLC mesophases of lipids intended as molecular switches for the “on-demand” release of chemicals, Small-Angle X-Ray Scattering (SAXS) has shown light-triggered effects. A tiny quantity of a lipid with an azobenzene photoactive unit that was synthesized successfully has been added to host liquid crystalline mesophases made up of oleic acid and monoolein.^40^ According to Jia et al, a promising class of photo-switchable molecules that hasn’t gotten much attention in the literature is hexaaryl biimidazoles. The one that exhibits remarkable negative photochromism and is sensitive to green light is (2,20-dimethoxy diphenyl imidazole)-1,10-binaphthyl.^41^ The photosensitive cubosomes utilized in the study by Bazylińska et al represent a substantial advancement in targeted therapy for melanoma skin cancer cells. These cubosomes, laden with photosensitizing dyes such as Chlorin e6 or meso- tetraphenylporphine-Mn (lll) chloride, exhibited enhanced biocompatibility, heightened therapeutic efficacy with significant cytotoxic effects post-irradiation effective bioimaging capabilities, and potential for future applications in photodynamic therapy and bioimaging of skin malignant melanoma. The innovative approach presents a promising avenue for precise and efficient treatment of melanoma skin cancer, underscoring the potential of cubosomes are versatile drug delivery systems for improved therapeutic outcomes.^42^

## Enzyme-responsive cubosomes

 One of the most promising types of smart stimulus-responsive NPs is enzyme-responsive NPs. Enzymes are needed for the body’s lipid processing to transform lipids into cellular fuel.^38^ The hepatoprotective properties and oral bioavailability of Coenzyme Q10 can be significantly enhanced by utilizing glycerol monooleate (GMO) cubosomes stabilized with P407. To overcome the challenges associated with piperine distribution, such as hydrophobicity and 1^st^ pass metabolism, tween 80-modified GMO cubosomes have been developed. *In vivo *studies indicate that these cubosomes markedly amplify cognitive function, suggesting their potential as a noninvasive, brain-targeted delivery system for Alzheimer’s disease treatment. Similarly, the incorporation of curcumin into phytantriol cubosomes has demonstrated a substantial increase in oral bioavailability, achieving at least a 14-fold improvement compared to free curcumin.^43^

 According to Tan et al cubosomes containing the antimicrobial peptide were found to have a notably greater bactericidal impact following enzyme exposure than pure peptide which lost its bactericidal action upon proteolysis.^43^ The significance of enzyme-sensitive cubosomes lies in their potential application as drug-delivery systems that can respond to specific enzymatic triggers. In the presence of enzymes present in the target tissue, the cubosomes can be synthesized for releasing payloads in a controlled fashion. This targeted and triggered release mechanism enhances the efficiency and specificity of drug delivery, especially in customized treatments of various diseases, hence offering precise dosage and minimal off-target effects. Fusion of enzyme-sensitive molecules and cubosomes can develop smart nanocarriers that react to specific biological cues such as enzyme activity levels in diseased tissues.^44,14^

 Based on the study conducted by Li et al, using enzymes as triggers, chemical selectivity and substrate specificity can be achieved. Enzyme-catalyzed reactions can be performed under moderate conditions, low-temperature aqueous environments, and neutral or near-neutral pH levels. Phospholipase exhibited high selectivity hydrolyzing fatty ester bonds at the sn-2 position of glycerophospholipids. Such cubosomes also increased the efficacy of urokinase-type plasminogen activator, a thrombolytic drug. Its encapsulation within the enzyme-responsive cubosomes aid in targeted drug release realized due to specific enzymatic triggers within the thrombus microenvironment. This ensures the thrombolytic agents retain protection while in circulation and are precisely released at the thrombosis site.^45^

## Multi stimuli responsive cubosomes

 Dual and multi-responsive, including stimuli-responsive NPs, are the innovative drug delivery strategies developed for combinational chemo-phototherapy. Integrating multiple stimuli - such as pH and redox, pH and temperature, temperature and magnetic field, enzyme activity, and others- resulted in multi-responsive drug delivery systems.For example co-loading photosensitizer and chemotherapeutic agents onto graphene oxide NPs has shown a marked improvement in cancer treatment efficacy compared to monotherapy.^46^ For targeted photodynamic treatment, a pH-responsive nanophotomedicine (pH-NanoPM) was developed. This nano photomedicine was constructed through the self-assembly of a pH-responsive polymeric photosensitizer (pH-PPS), incorporating approximately 10nm-sized pH-cleavable mPEG (pH-pH-mPEG). When HeLa human cervical cancer cells were exposed to pH-NanoPM, enhanced cellular internalization was observed at the acidic tumor pH compared to the normal pH, leading to a significant increase in cancer cell cytotoxicity. The fusion of metal NPs and stimuli-responsive polymers onto one platform has garnered a lot of attention in recent years. Zhou et al stated that a drug in combination with a polymer containing selenium may be employed successfully for multi-stimuli responsive drug release. They created metal-organic frameworks with pH-triggered properties for drug delivery systems and selenium-containing PEG micelles with redox-triggered features. It is observed that the shell can only break down in low pH conditions, the cores collapsed readily in the presence of redox agents.^47^

 As reported by Sauraj et al in their study on pH-sensitive prodrug NPs for targeted chemo-photodynamic therapy, the integrated platform was formed by encapsulating the photosensitizer after connecting the chemotherapeutic agent DOX to the polymer PEG via a pH-sensitive (Schiff base) bond. Under acidic pH conditions, the NPs exhibited pH-responsive release behavior, leading to the simultaneous release of the medication and photosensitizer. An *in vivo *investigation revealed that NPs had higher antitumor efficacy against the cells when compared to free drugs and photosensitizers.^46^ Details on types of cubosomes and their potential applications are given in Table 1.

**Table 1:** Types of responsive cubosomes and their potential applications

**Types of responsive cubosomes**	**Composition**	**Drug used**	**Applications**	**References**
pH-responsive	Monoolein Pluronic F127 Ionizable amino lipids	Doxorubicin	Anticancer Therapy	Rajesh et al^48^
Light-responsive cubosomes	Photoswitchable amphiphiles such as azobenzene photo surfactants and monoolein	Nile Red	Controlled drug delivery.	Jones et al^49^
pH-responsive cubosomes	Monoolein and the amino lipids N-(Pyridin-4-ylmethyl) oleamide and N-(2(piperidine-1yl)ethyl) oleamide	7-ethyl-10-hydroxy camptothecin, which is an active metabolite of the anticancer prodrug irinotecan	Anticancer Therapy	Rajesh et al^29^
pH-responsive cubosomes	Monoolein N-arginine-modified chitosan and alginate	Anthelmintic drugs including ivermectin, mebendazole, and praziquantel 1.	oral drug delivery systems	Mathews et al^28^
Cubosomes in thermos responsive gelling system	Glyceryl monooleate, Pluronic® F127,	Docetaxel	Controlled-release	Rarokar et al^50^
Thermosensitive cubosomes	Poloxamer 407 Glyceryl monooleate	Lamotrigine	intranasal delivery	Mohsen et al ^34^
Thermosensitive cubosomes	poly(N,N-dimethyl acrylamide)-block-poly(N-isopropyl acrylamide) glycerol-monooleate	-	targeted drug delivery	Balestri et al ^51^
pH-responsive cubosomes	Monoolein. Brucea javanica oil	Doxorubicin	combined delivery for cancer treatment	Li et al^52^

## Top-down and bottom-up approaches for cubosomes preparation

 In the bottom-up approach, cubosomes are fashioned through the dispersion of droplets from the inverse micellar phase into an aqueous medium heated to 80 °C. subsequently, a gradual cooling process ensues, prompting crystallization and the emergence of cubosomes. The hydrotrope incorporated within the cubosomes formulation assumes a pivotal role in thwarting the development of a bulk cubic gel phase. Its action involves a dissolution of the cubic gel, while the subsequent introduction of water, in conjunction with sonication, diminishes the solubility of the liquid crystalline particles, thus fostering the genesis of cubic entities.^53^

 According to Gaballa et al, poloxamer 407 and soulan C24 were used as stabilizers throughout the top-down process of creating GMO cubosomes.^54^ As per Garg et al, the surfactant employed in the production of cubosomes includes poloxamer 407, with the concentration ranging from 0%-20% w/w about the dispersion phase. Typically, a concentration of 2.5%-10% w/w of the total weight of the dispersion is necessary for the monoglyceride/surfactant mixture. In addition to poloxamer, polyvinyl alcohol (PVA) is utilized as a dispersion stabilizer.^55^ According to Bryant et al phytantriol solutions in a variety of diluents, such as glycerol, ethanol, honey, lactic acid, and choline chloride-glycerol, were used to create cubosomes. Applying these solutions dropwise to water containing poloxamer 407 stabilizers was done following a well-established cubosome synthesis protocol.^56^ According to Gaballa et al to avoid cubosome dispersion aggregation, F127 or another appropriate stabilizer must be used. The selection of the ideal preparation technique still focuses primarily on stability, biocompatibility, and optimal drug release.^57,58^

## Challenges and considerations

 Addressing the potential challenges inherent in cubosomes production and stability is paramount to fully harnessing their potential in pharmaceutical applications. A significant obstacle is the elevated viscosity of the cubic phase, which complicates large-scale production processes. Moreover, cubosomes tend to exhibit low entrapment efficiency for water-soluble drug molecules due to the significant water content within their structure. This constraint not only has an impact on overall medication loading capacity, but it also impairs the delivery system’s effectiveness. Nano-sized nature of cubosomes can undergo particle growth upon prolonged standing and is problematic for parenteral formulations. This causes stability and uniformity issues, demands innovative approaches to mitigate particle proliferation, and assures long-term stability.^24,59-61^

 There is continuous research on cubosomes in drug delivery aimed at surmounting production limitations and augmenting stability. Researchers hope to unlock the complete potential of cubosomes by tackling difficulties associated with cubosomes such as viscosity maintenance during the synthesis and refinement of drug-loading methods for hydrophilic compounds. The NP engineering and tailored formulations promise to mitigate stability concerns connected to particle growth, thereby enhancing the suitability of cubosomes for various administration routes including parenteral delivery. The challenges can be resolved with interdisciplinary research efforts, shaping the future landscape for customized drug delivery options.^22,62-64^

## Diverse utilization

 Cubosomes have emerged as a promising drug delivery system, presenting advantages over traditional liposomes because of their unique inner cubic structure. This unique configuration provides a significantly larger interfacial surface area, facilitating the encapsulation and protection of higher quantities of hydrophilic and hydrophobic drugs compared to liposomes.^65-66,14^ Notably, cubosome preparation primarily employs shear and homogenization techniques, eliminating the need for organic solvents. Furthermore, cubosomes exhibit superior solubilization capacities in contrast to conventional lipid or non-lipid carriers, making them excellent vehicles for protecting delicate drugs, such as peptides and proteins, from enzymatic degradation and *in vivo* degradation.^67^ Nanocarriers, including cubosomes, exhibit minimal toxicity and biocompatible characteristics, making them effective delivery methods for a wide range of substances in various applications.^68-70,26^

 Cubosomes have shown promise as nanocarriers for anticancer medications. The unique structure of cubosomes suggests their potential application in melanoma treatment, with both passive and active targeting strategies demonstrating validity in preclinical and clinical research. In the realm of oral drug delivery, liquid crystalline NP technology emerges as a sophisticated solution, adept at orchestrating precise *in vivo* drug distribution. By strategically releasing particles at distinct absorption sites, such as the upper or lower intestine, it effectively navigates the challenges of regional absorption, a critical consideration for medications characterized by narrow absorption windows.^71-75^

 In targeted drug delivery, cubosomes have exhibited enhanced permeability and retention when administered to rabbit corneal tissue sections, showcasing their potential for ocular applications. Additionally, cubosomes have been found to increase ocular bioavailability by prolonging the half-life at the corneal surface and exhibiting mucoadhesive properties, enhancing corneal permeability. For topical drug delivery, the bio-adhesive characteristics of cubic phases make them suitable for mucosal depositions and topical drug delivery systems. These systems leverage liquid crystal and liquid crystal NP technology to create bio-adhesive liquid crystalline systems in situ, facilitating precise and efficient drug distribution to mucosal surfaces. In contrast to conventional administration approaches, topical drug delivery systems offer temporary protection to sensitive and irritated skin by creating a thin layer on mucosal surfaces. These systems further fine-tune the nanostructure to attain the desired delivery profiles, representing a sophisticated approach to dermatological care.^48,76^

## Conclusion

 Cubosomes demonstrate strong potential as smart drug delivery systems, owing to their ability to respond to physiological stimuli such as temperature, pH, and enzymatic activity. This review presents that cubosomes support the initial hypothesis of their functional adaptability since they show great potential as carriers for site-specific and controlled drug delivery. Their distinctive structural characteristics and biocompatibility imply useful benefits in improving drug stability and release profiles. By minimizing off-target effects and enhancing delivery precision, cubosomes could contribute meaningfully to the development of safer and more effective therapies. Future studies may centre on optimizing formulation parameters, increasing manufacturing scale, and conducting in vivo studies to support their clinical relevance in particular therapeutic settings.

## Competing Interests

 None.

## Ethical Approval

 Not applicable.
